# Genetic Response of Common Bean to the Inoculation with Indigenous *Fusarium* Isolates

**DOI:** 10.3390/jof6040228

**Published:** 2020-10-16

**Authors:** Alejandra J. Porteous-Álvarez, Sara Mayo-Prieto, Samuel Álvarez-García, Bonifacio Reinoso, Pedro A. Casquero

**Affiliations:** Grupo Universitario de Investigación en Ingeniería y Agricultura Sostenible (GUIIAS), Instituto de Medio Ambiente, Recursos Naturales y Biodiversidad, Universidad de León, Avenida Portugal 41, 24071 León, Spain; apora@unileon.es (A.J.P.-Á.); smayp@unileon.es (S.M.-P.); salvg@unileon.es (S.Á.-G.); breis@unileon.es (B.R.)

**Keywords:** biocontrol, biodiversity, legume, *Phaseolus vulgaris*, qPCR, soil-borne pathogens

## Abstract

Fungal species from the genus *Fusarium* are important soil-borne pathogens worldwide, causing significant economic losses in diverse crops. The need to find sustainable solutions against this disease has led to the development of new strategies—for instance, the use of biocontrol agents. In this regard, non-pathogenic *Fusarium* isolates have demonstrated their ability to help other plants withstand subsequent pathogen attacks. In the present work, several *Fusarium* isolates were evaluated in climatic chambers to identify those presenting low or non-pathogenic behavior. The inoculation with a low-pathogenic isolate of the fungus did not affect the development of the plant, contrary to the results observed in plants inoculated with pathogenic isolates. The expression of defense-related genes was evaluated and compared between plants inoculated with pathogenic and low-pathogenic *Fusarium* isolates. Low-pathogenic isolates caused a general downregulation of several plant defense-related genes, while pathogenic ones produced an upregulation of these genes. This kind of response to low-pathogenic fungal isolates has been already described for other plant species and fungal pathogens, being related to enhanced tolerance to later pathogen attacks. The results here presented suggest that low-pathogenic *F. oxysporum* and *F. solani* isolates may have potential biocontrol activity against bean pathogens via induced and systemic responses in the plant.

## 1. Introduction

Common bean (*Phaseolus vulgaris* L.) is one of the most important legume crops worldwide, being the third most produced legume after soybean (*Glycine max* (L.) Merr.) and peanut (*Arachis hypogea* L.). The harvested area of beans has steadily increased in the last few decades, from 23 million hectares in 1999 to 36 million hectares in 2017. Similarly, the production increased from 15 million tons in 1987 to over 31 million tons in 2017. In Europe, Spain is the ninth country in terms of production, with 19,675 tons in 2017 [1]. León is a region located in the northwest of Spain and the main common bean producer in Spain both in quality and quantity, with almost 60% of the national production in 2017 [2]. The Protected Geographical Indication (PGI) “Alubia La Bañeza-León,” (PGI-ALBL) (EC Reg. n. 256/2010 published on 26 March 2010, OJEU L880/17) shelters the high quality of this product. This PGI includes four different landraces: “Riñón”, “Pinta”, “Canela”, and “Plancheta”.

*P. vulgaris* as a legume crop is very treasured as it is capable, due to its association with some bacterial species of the genus *Rhizobium*, of fixing nitrogen to the soil [3], improving its absorption even in subsequent crops. Common bean crops are affected by a variety of pests and diseases caused by bacteria, fungi, and viruses, some of them resulting in important economic losses [4,5,6,7]. One of the fungi affecting this and many other crops is *Fusarium* spp. [8], a filamentous fungus that belongs to the phylum *Ascomycota*. Two main species from this genus cause large losses in common bean crops, *F. solani* (Mart.) Sacc. f. sp. *phaseoli* (Burkholder) W. C. Snyder & H. N. Hans and *F. oxysporum* Schlechtend: Fr. f. sp. *phaseoli* J. B. Kendrick & W. C. Snyder.

Characteristic symptoms of the presence of *F. solani* f. sp. *phaseoli* are narrow longitudinal red to brown patches in the hypocotyl and main root during the early days after germination. Over time, these patches turn necrotic, and infected plants grow adventitious roots at surface level [6], which allows the plant to recover some of its water and nutrient absorption capacity [9]. Crop damage intensifies under conditions of soil compaction, drought, or reduced root growth. Diseased plants present diverse sizes within the field, which affects productivity [6].

*F. oxysporum* f. sp. *phaseoli* cause tracheomycosis. The symptoms observed in the plant are a yellow coloration and premature senescence in lower leaves. As the disease advances, these symptoms are evident in upper parts of the plant. The vascular system presents a reddish-brown color [6] even before showing yellowing or wilt in the leaves [9].

Current management of these diseases in the field relies on chemical seed treatment, other localized treatments, or cultural practices. These strategies have not completely solved the problem, as the use of chemicals is rarely cost-effective [6,8] and cultural practices help to prevent but not to eradicate the infestation. The need to find new sustainable and environment-friendly solutions against these pathogens has led to the search for biocontrol agents belonging to genera such as *Agrobacterium, Bacillus, Gliocladium*, or *Trichoderma* [10], non-pathogenic strains of the same phytopathogenic species, or other organisms that can help the plant overcome the attack [11]. Plants respond to different biotic and abiotic stresses by several defense mechanisms: constitutive, induced, or acquired. Constitutive defenses are present in the plant before the attack or stress and they can be either physical or biochemical mechanisms [12]. The induced defense is triggered during the infection by a pathogen in the localized area. The hypersensitive response, an induced defense mechanism, can entail cellular death, controlling the advance of the pathogen within the plant [13]. A systemic acquired response (SAR) is triggered after the attack of a pathogen and activates signaling pathways by the overexpression of defense-related genes that help the plant stand the attack before coming into contact with the pathogen [14]. Some of the molecules responsible for induced and systemic responses are salicylic acid, jasmonic acid, ethylene, or reactive oxygen species (ROS).

A non-pathogenic strain is unable to produce disease on a given plant species even though they colonize tissues of the plant [15]. However, the interaction with a non-pathogenic organism can lead to the activation of induced systemic resistance (ISR) in the plant [16,17]. In this regard, induction of the host plant resistance by non-pathogenic isolates has been demonstrated for several pathogens such as *Rhizoctonia* spp., *Fusarium* spp., and *Pythium* spp. in numerous crops [18]. There are some studies where the presence of non-pathogenic fungal isolates protects plants against pathogenic isolates of the same fungus, different fungal species, or even other organisms. The application of a non-pathogenic isolate of *F. oxysporum* showed protection in cucumbers against *Pythium ultimum* [19], in bananas against the nematode *Radopholus similis* [20], or in the tomato against a pathogenic isolate of the same species [11]. Treatment of *P. vulgaris* with *Pseudomonas putida* in the soil reduced aerial disease caused by *Botrytis cinerea* [21].

This study aims to analyze and compare the expression of *P. vulgaris* defense-related genes when inoculated with indigenous pathogenic and low-pathogenic isolates of *Fusarium*. The hypothesis of the present work is that the expression pattern of *P. vulgaris* defense-related genes differs in response to the exposition to pathogenic and low-pathogenic Fusarium isolates, the latter being more similar to those responses triggered by biocontrol agents than by phytopathogenic fungal isolates.

## 2. Materials and Methods

### 2.1. Fusarium spp. and Culture Collections

The present study was conducted using one isolate of *F. solani* and eleven isolates of *F. oxysporum*, collected from symptomatic bean plants [22] in the production area of PGI-ALBL (Table 1). All fungal isolates were kept in the “Pathogens and Antagonists of the Laboratory Diagnosis of Pests and Diseases” collection (PALDPD, University of León). One week prior to their use, all isolates were activated by culturing them on potato-dextrose-agar medium (PDA, Sigma-Aldrich, St. Louis, MO, USA) at 22 °C.

A Nucleospin Plant II kit (Macherey-Nagel, Düren, Germany) was used to extract the genomic DNA from 100 mg of each fungal isolate, following the manufacturer’s protocol for fungi. The resulting extracts were eluted in 50 μL of sterile water and a NanoDrop ND-1000 Spectrophotometer (Thermo Scientific, Wilmington, DE, USA) was used to estimate the DNA concentration. Solutions of 50 μL containing 10 mM Tris-HCl (pH 8.3), 50 mM KCl, 1.5 mM MgCl_2_, 1.5 U of DreamTaq DNA polymerase (Thermo Scientific), 200 M for each dNTP, 400 nM for each primer, and 50 ng of DNA were used to amplify the sequences. A fragment of the TEF1 and ITS5-ITS4 were used to amplify nuclear rDNA-TEF1 and rDNA-ITS regions. PCR products were purified using the NucleoSpinExtract II kit (Machery-Nagel, Düren, Germany), sequencing them afterwards using a fragment of TEF1 and ITS4 as primer and the BigDye Terminator v3.1 Cycle Sequencing Kit (Applied Biosystems, Foster City, CA, USA) with an automatic capillar sequencer ABI 3130xl (Applied Biosystems). All the steps were performed in accordance with the manufacturer’s instructions. In order to identify the fungal isolates, the sequences obtained were analyzed and compared with those of the NCBI Genbank database (National Center for Biotechnology Information, http://www.ncbi.nlm.nih.gov) using the BLAST tool (http://www.ncbi.nlm.nih.gov/BLAST).

### 2.2. In Vivo Assay

The bioassays were performed in growth chambers: control without fungi (CC), eleven *F. oxysporum* isolates (F1–F12), and one *F. solani* isolate (F13) (Table 1). Two separate sets of fifteen polypropylene pots (1-L capacity) with non-autoclaved substrate (80% white peat, 20% black peat and pH 5.5) were used per treatment.

Each pot was watered with 250 mL and the fungi were inoculated by surface irrigation with a suspension of a blended fungal culture of each isolate (50 mL/pot). This suspension was the result of mixing five Petri dishes containing 7-day-old colonies of the corresponding fungal isolate grown on 15 mL of PDA per liter of distilled water. A similar suspension using only PDA medium served as control. Pots were kept in a growth chamber for 8 days at 25 °C (16 h) and 16 °C (8 h), 60% relative humidity (RH) in the dark until sowing.

“Canela” bean seeds (PGI-ALBL) were surface disinfected with sodium hypochlorite 1% for 3 min and then washed for 6 min in distilled water. Eight days after the previously described fungal inoculation, two beans were sown per pot, accounting for a total of sixty seeds per treatment.

The plants were maintained for 60 days with a photoperiod of 16:8 h light:darkness, a temperature of 25 °C/16 °C day/night, 60% RH, and brightness of 3500 lux. Irrigation was performed every 4 days with tap water (250 mL/pot). Every 2 weeks, a nutrient solution was added (250 mL/pot) [23]. Plants were removed 60 days after sowing (DAS). Wet and dry weight (72 h 80 °C) of the aerial part and root system of the sixty plants were evaluated. Based on the results obtained, two low-pathogenic and one pathogenic *Fusarium* were selected for further assays.

In order to ensure Koch’s postulates, hypocotyls were surface disinfected by submersion in 1% sodium hypochlorite for 1 min and were washed for 3 min in distilled water. Afterwards, they were cut into sections and placed on Rose Bengal chloramphenicol agar medium (Conda Laboratory, Torrejón de Ardoz, Madrid, Spain) and kept at 22 °C for one week to re-isolate and identify the fungi.

All data were compared by analysis of variance (ANOVA) after confirming normality and equality of variances and were compared by Fisher least significant difference (LSD) post hoc tests using SPSS (IBM SPSS Statistics for Windows, Version 24.0. Armonk, NY, USA).

### 2.3. RNA Extraction and Purification

**Figure 1:**
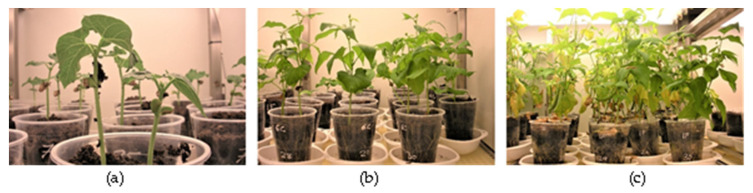
Bean sampling stages. (**a**) 14 DAS, (**b**) 35 DAS, and (**c**) 56 DAS.

Three leaves from three different plants were collected from each treatment at three different times, namely when first leaves appeared (14 DAS), when trifoliate leaves appeared (35 DAS), and when first pods appeared (56 DAS) (Figure 1). Leaves were stored at −80 °C for further analyses.

RNA extraction was performed as previously described by Mayo et al. [24]. The A260/280 absorbance ratio was measured with a NanoDrop 2000 (Thermo Scientific, Wilmington, DE, USA) to evaluate RNA concentration and purity, considering 1.8 ≤ A260/280 ≤ 2.0 as the ideal absorbance ratio. Finally, the integrity of the RNA was evaluated by electrophoresis, running the samples in a 1% agarose gel.

### 2.4. cDNA Synthesis

DNA was removed from the samples using a TURBO DNA free^TM^ kit (Applied Biosystems, Foster City, CA, USA) in accordance with the manufacturer’s instructions. cDNA was synthesized from 1 µg of total RNA using a Reverse Transcription System with an Oligo(dT)_15_ as primer (Promega, Madison, WT, USA) and following the manufacturer’s instructions. Finally, cDNA was quantified using a Nanodrop 2000.

### 2.5. Real Time-PCR Analysis

A PCR was performed to evaluate the expression of twenty genes, which were selected for their expression in common bean leaves described by previous studies [24,25] (Table 2). The reactions were carried out in a total volume of 25 μL: 2.5 μL Taq buffer 10X, 2.5 μL dNTPs 2 mM, 1.75 μL MgCl_2_ 25 mM, 0.5 μL forward primer 20 μM, 0.5 μL reverse primer 20 μM, 0.25 μL Taq Polymerase (Fermentas), 2.5 μL cDNA, and H_2_O Milli-Q^®^ to 25 μL. For the negative control, no cDNA template was added. The thermal cycler program was initiated by preheating the reaction mixture to 95 °C for 10 min, then 40 cycles of 95 °C for 15 s and 60 °C for 15 s. A gel electrophoresis was carried out to confirm PCR amplification. The genes amplified in the PCR were subsequently analyzed by qPCR.

qPCR reactions were carried out using Step One Plus™ (Applied Biosystems, Foster City, CA, USA). These reactions took place in a total volume of 20 μL, consisting of 0.3 μL of forward primer 20 μM, 0.3 μL of reverse primer 20 μM, 1 μL of cDNA, 10 μL of Power SYBR^®^ Green PCR Master Mix (Applied Biosystems, USA), and H_2_O Milli-Q^®^ to complete the 20 μL. Each reaction was performed in triplicate. The already described cycling parameters were applied to run the reactions [26] and the resulting data were analyzed using the 2^−ΔΔCt^ method [27].

To determine the relative expression level of each gene, *UKN1* and *Act11* [28] were used as housekeeping genes. The control treatment served as a reference to compare the *Fusarium* treatments.

## 3. Results

### 3.1. In Vivo Assay: Selection of Pathogenic and Low-Pathogenic Fusarum Isolates

**Figure 2:**
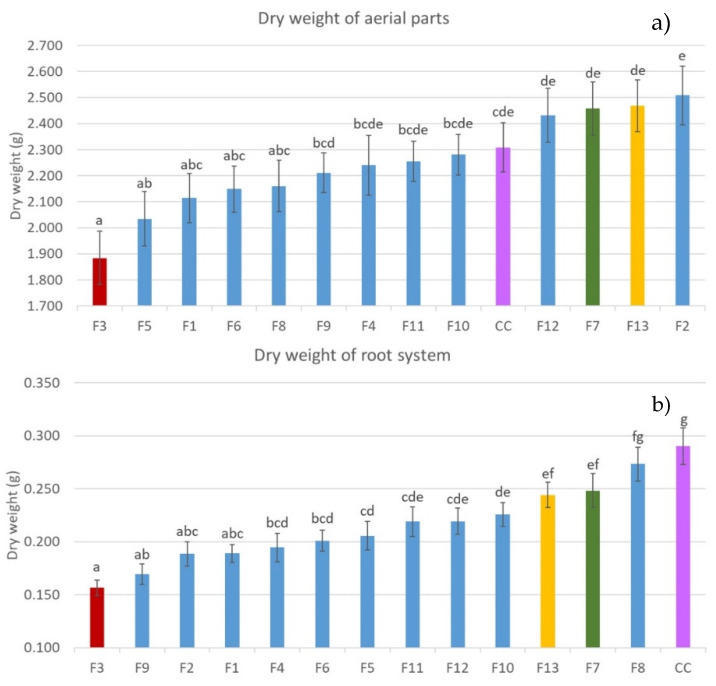
Dry weight of the aerial parts ± SD (standard deviation bar) (**a**) and root system (**b**) of 60-day-old plants. *F. oxysporum* (F1–F12), *F. solani* (F13), and control without fungus (CC). Values are means ± SE of 30 plants. Columns with different colors indicate selected treatments for further assays; CC (purple), *F. oxysporum* F3 (red), *F. oxysporum* F7 (green), and *F. solani* F13 (yellow). The data were compared by analysis of variance (ANOVA) and Fisher least significant difference (LSD). Different letters mean statistically significant differences (*p* < 0.05) between treatments.

Regarding the dry weight of the aerial part, plants grown in the presence of *F. oxysporum* F2, F7, and F12, and *F. solani* F13, showed a higher dry weight than the control (CC), but without significant differences from it. On the other hand, the referred treatments showed significantly higher dry weight than plants grown in the presence of *F. oxysporum* F1, F3, F5, and F6 isolates. Out of these four isolates, only F3 and F5 presented significant differences from the control, the former being the one producing the lowest aerial dry weight (Figure 2).

Regarding the plants’ root system, all treatments showed significantly less dry weight than the control. Plants grown on a substrate inoculated with *F. oxysporum* F7 and *F. solani* F13 showed the smaller reduction in root weight, being less than 25% with respect to the control. Treatments with F3 and F9 (*F. oxysporum*) yielded the lowest dry root weight compared to the control (Figure 2).

Based on the abovementioned results, *F. oxysporum* F3 was selected for further studies as the most pathogenic of the tested isolates. On the other hand, *F. oxysporum* F7 and *F. solani* F13 were equally selected for their low-pathogenic behavior. These two isolates showed the least significant differences to the control treatment in both aerial part and root system dry weight, while showing at the same time significantly higher values in both parameters compared to the most pathogenic isolate, *F. oxysporum* F3. The fact that F7 and F13 did not affect the plant’s aerial development, even though they reduced its root system weight, is a strong indicator of the reduced pathogenicity of these isolates.

### 3.2. Re-Isolation of the Fungal Isolates from Infected Plants

**Figure 3:**
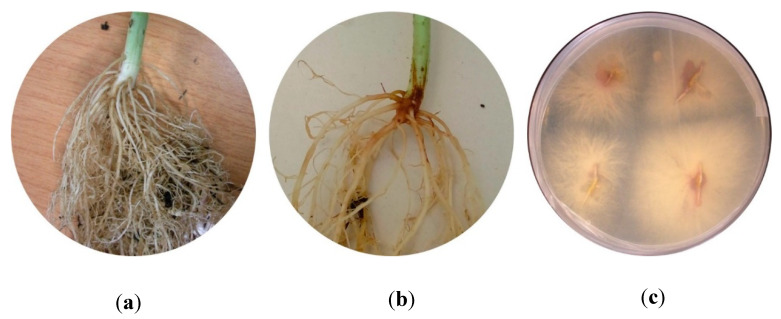
Bean symptoms in hypocotyl and root system of (**a**) control treatment and (**b**) infected plant. (**c**) Petri plate with infected hypocotyls showing *Fusarium* growth (re-isolation).

To ensure Koch’s postulates, hypocotyls were placed on Rose Bengal medium after plants were extracted 60 DAS. All treatments, with the exception of the controls, presented *Fusarium* growth on the medium, thus confirming the presence of the isolates in the bean plants (Figure 3). The identification of the isolates was performed by visual and microscope comparison.

### 3.3. Expression of Bean Defense-Related Genes

In this study, the expression of twenty defense-related genes was evaluated and compared between plants exposed to the described pathogenic and low-pathogenic *Fusarium* isolates. This expression was analyzed in leaves at three different stages of development, 14, 35, and 56 DAS, corresponding to the appearance of the first leaves, the first trifoliate leaves and the first pods, respectively (Figure 1). These genes were selected according to previous studies [24,25] and detection of expression in bean leaves.

**Figure 4:**
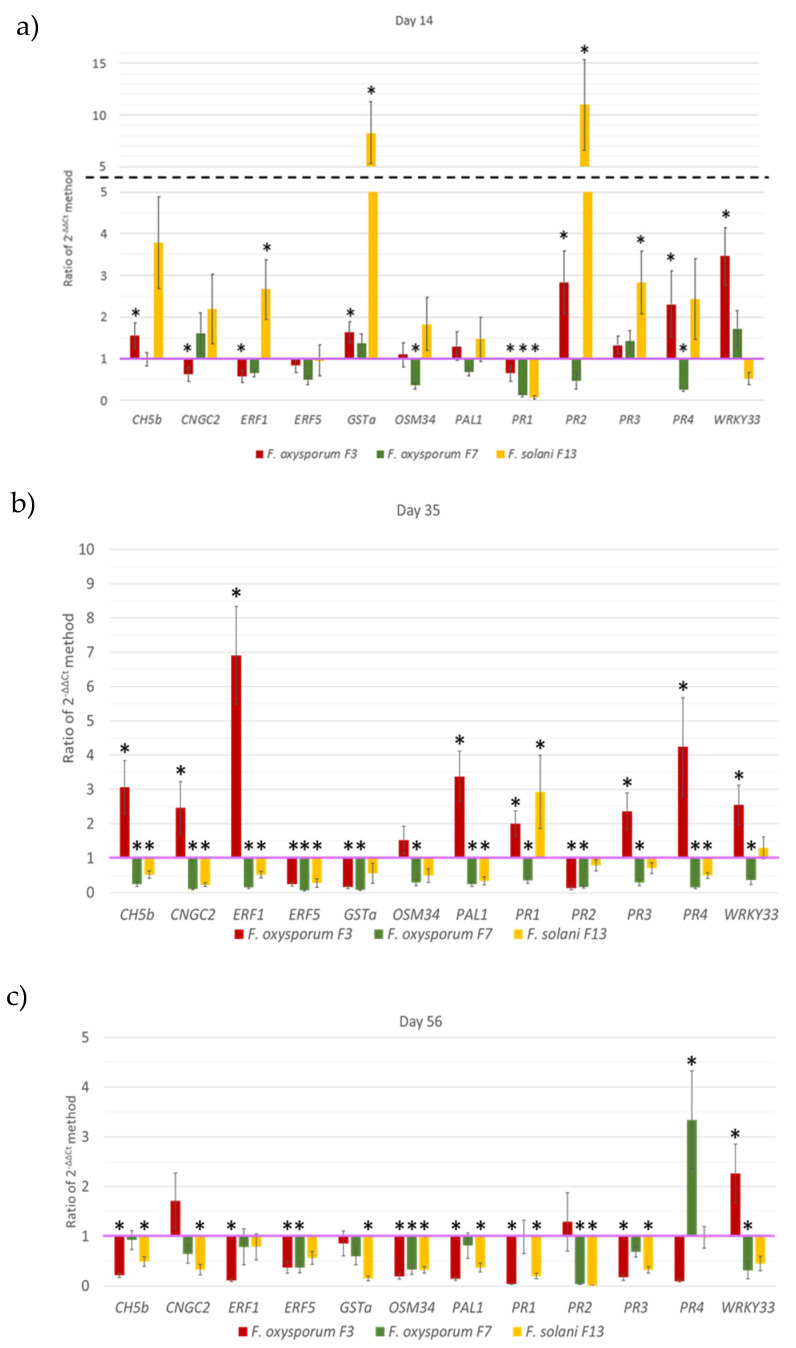
Analysis of relative expression levels of the bean defense-related genes in (**a**) 14, (**b**) 35, and (**c**) 56-day-old bean plants treated with pathogenic *F. oxysporum* F3 (red), low-pathogenic *F. oxysporum* F7 (green), and low-pathogenic *F. solani* F13 (yellow) compared to control plants. The purple line represents the expression level of each gene in control plants. Values are 2^−ΔΔCt^ ± SD (standard deviation bar) of 3 measurements. The data were analyzed by the 2^−ΔΔCt^ method. An asterisk (*) indicates statistically significant differences (*p* < 0.05) between the gene expression of treated and control plants.

A PCR was performed to confirm the expression of twenty defense-related genes in bean leaves. Those genes with low expression levels were discarded, leaving a total of fourteen genes to be analyzed by qPCR. Out of these fourteen, twelve showed reliable results in qPCR analysis (Figure 4) and therefore were selected for further study. *UKN1* was used as the only housekeeping gene because *Act11* showed no stable qPCR results.

In the first stage, 14 DAS (Figure 4a), plants treated with *F. oxysporum* F3 showed significant downregulation in the genes *CNGC2*, *ERF1*, and *PR1* while *CH5b*, *GSTa*, *PR2*, *PR4*, and *WRKY33* presented significant upregulation. On the other hand, plants treated with *F. oxysporum* F7 showed significant downregulation in the genes *OSM34*, *PR1*, and *PR4*, while the rest of them showed no significant differences to the control plants. Besides, plants treated with *F. solani* F13 inoculated substrate showed significant upregulation of genes *CH5b*, *ERF1*, *GSTa*, *PR2*, and *PR3*, while *PR1* was downregulated.

In the second stage, 35 DAS (Figure 4b), plants treated with *F. oxysporum* F3 showed significant downregulation in the genes *ERF5*, *GSTa*, and *PR2* while the genes *CH5b*, *CNGC2*, *ERF1*, *PAL1*, *PR1*, *PR3*, *PR4*, and *WRKY33* showed significant upregulation. On the other hand, plants grown on the substrate inoculated with *F. oxysporum* F7 showed significant downregulation in all genes analyzed, and plants treated with *F. solani* F13 had significant upregulation of *PR1* and downregulation of the genes *CH5b*, *CNGC2*, *ERF1*, *ERF5*, *PAL1*, and *PR4*.

In the last stage, 56 DAS (Figure 4c), plants treated with *F. oxysporum* F3 showed significant downregulation in the genes *CH5b*, *ERF1*, *ERF5*, *OSM34*, *PAL1*, *PR1*, *PR3*, and *PR4*, while *WRKY33* presented significant upregulation. On the other hand, plants treated with *F. oxysporum* F7 showed upregulation of the *PR4* gene and downregulation of *ERF5*, *OSM34*, *PR2*, and *WRKY33*. Finally, plants treated with *F. solani* F13 presented no significant upregulation in any analyzed genes, showing significant downregulation of *CH5b*, *CNGC2*, *GSTa*, *OSM34*, *PAL1*, *PR1*, *PR2*, and *PR3*.

## 4. Discussion

In the present study, plants grown on a substrate inoculated with *F. oxysporum* F7 and *F. solani* F13 isolates showed similar behavior to that described by Ting et al. [29], who observed that plants had no significant differences when inoculated with non-pathogenic isolates of *F. oxysporum* compared to control treatment with water, while the inoculation of a pathogenic isolate showed an inhibited plant growth. These results allow us to identify the referred fungal isolates as low-pathogenic, but not entirely non-pathogenic, as they significantly reduced root development. Nevertheless, this effect on the roots did not affect the aerial growth of the plants, indicating that their overall development was not deeply affected.

As it is well known, plants respond to different stresses by several physical or biochemical mechanisms [12,13]. WRKY transcription factors are involved in multiple biological functions regarding plant resistance to diseases, nutrient deprivation, abiotic stresses, as well as seed and trichome development, senescence, embryogenesis, and other developmental and hormone-controlled processes [24,30]. In *Arabidopsis*, *WRKY33* has a role in biotic stress defense, where it regulates the balance between necrotrophic and biotrophic pathogen responses [31,32,33]. *WRKY* family members are responsible for the regulation of the expression of *PR* genes in common bean and *Arabidopsis* [24,33]. *PR1*, *PR2*, *PR3*, and *PR4* are pathogenesis-related genes with diverse functions. Mayo et al. [24] observed that 45 DAS, significant downregulation of *WRKY33* and the *PR* genes took place when the bean plants grew on a substrate inoculated with both a phytopathogenic fungal isolate and a biocontrol agent, as well as when only the biocontrol agent was applied. Another study demonstrated that when *Arabidopsis thaliana* was inoculated with necrotrophic fungus *B. cinerea*, the upregulation of *WRKY33* affected the expression of *PR* genes negatively and enhanced plant resistance to the fungus. In contrast, when plants were healthy or were not infected, *WRKY33* was expressed at low levels [33]. In the present study, bean plants treated with *F. oxysporum* F7 35 DAS or with *F. solani* F13 56 DAS showed downregulation of these genes without significant differences from the control. Conversely, plants grown on the substrate inoculated with *F. oxysporum* F3, 35 DAS, showed significant upregulation of *WRKY33* and the *PR* genes analyzed, except for *PR2*, which was downregulated. Moreover, in plants treated with the same pathogenic isolate and analyzed 56 DAS, *WRKY33* was upregulated but the *PR* genes were significantly downregulated. These differences in the plant defense response suggest that the low-pathogenic fungi trigger a defense response different to that triggered by the pathogenic one.

*CH5b*, *ERF1*, and *ERF5* are genes related to the ethylene signaling pathway. *CH5b* encodes an endochitinase precursor [24]; *ERF1* and *ERF5* in *Arabidopsis* are ethylene response factors, components of the ethylene and jasmonic acid defense responses [34]. Mayo et al. [24] reported significant downregulation of *ERF1* and *ERF5*, 45 DAS, when bean plants were grown on a substrate inoculated with either a pathogen or a biocontrol agent, but *CH5b* showed no significant differences to control plants when inoculated with a pathogen, while showing upregulation when inoculated with a biocontrol agent. Boller [35] affirmed that chitinase expression level is low in healthy or uninfected plants, while its expression is enhanced under pathogen attack or ethylene treatment. In this work, plants reacted to treatment with *F. oxysporum* F3 and *F. solani* F13 as if they were pathogens, showing upregulation of *CH5b* in the first stage. Nevertheless, plants exposed to *F. solani* F13 readjusted this *CH5b* expression below that of control plants from 35 DAS onwards.

*CNGC2* (cyclic nucleotide-gated ion channel 2) is related to the Ca^2+^ influx and K^+^ and Cl^−^ efflux. It can interact in the initiation of the programmed cell death in *Arabidopsis* [36]. This gene was downregulated in bean plants treated with a biocontrol agent, *T. velutinum*, 45 DAS [24]. In the present study, plants inoculated with *F. oxysporum* F3 showed significant upregulation of *CNGC2* 35 DAS. On the contrary, they showed significant downregulation of this gene during the same stage when inoculated with *F. oxysporum* F7 and presented a similar response 35 and 56 DAS when inoculated with *F. solani* F13. These latter results agree with those obtained by Mayo et al. [24] when a biocontrol agent was inoculated to the bean plants, which may suggest that the plant identifies the low-pathogenic fungi as harmless, thus triggering a response closely related to that set off by biocontrol agents and qualitatively different from that exerted by phytopathogenic isolates.

*GSTa* (2,4-D inducible glutathione S-transferase) responds to pathogenic attacks [37] and can be induced by salicylic acid, methyl jasmonate, abscisic acid, and H_2_O_2_ [38,39]. One of its roles is related to the detoxification of harmful substances, attenuation of oxidative stress, and participation in hormone transportation [40]. Different plants treated with beneficial organisms often show an induction in the expression of *GST* genes which may be related to the induced systemic resistance in the plant [40]. In this case, this gene is strongly upregulated in plants treated with *F. solani* F13 14 DAS.

Osmotins have a protective effect against biotic and abiotic stresses and can play a role in the regulation of metabolic energy [41]. *OSM34* encodes an osmotin-like protein. Osmotins show antifungal activity when overexpressed, being able to inhibit spore germination, hyphal growth, producing spore lysis, or reduce the viability of the spores [42]. Mayo et al. [24] observed a downregulation of this gene when the pathogen and a biocontrol agent were present, while when the pathogen or the biocontrol agent were alone, the expression of the *OSM34* gene showed no significant differences to control plants. In the present work, no significant upregulation of the gene was observed at any stage or treatment, showing significant downregulation for all treatments 56 DAS.

Finally, *PAL1* (phenylalanine ammonia-lyase) is involved in the biosynthesis of salicylic acid. The presence of a pathogenic *Rhizoctonia solani* resulted in an upregulation in the expression of this gene, while the presence of a non-pathogenic *R. solani* produced its downregulation in *P. vulgaris* [43]. Significant downregulation of its expression was also observed in *P. vulgaris* when plants grew in contact with the biocontrol agent *T. velutinum* [24]. In banana, Paparu et al. [20] observed a non-statistically significant downregulation of the expression of the gene 33 days after inoculation of a non-pathogenic isolate of *F. oxysporum*. These results agree with the obtained in this study, in which *PAL1* showed upregulation 35 DAS in plants treated with *F. oxysporum* F3, while showing downregulation 35 DAS in plants treated with *F. oxysporum* F7, and 35 and 56 DAS in plants treated with *F. solani* F13.

As it has been reported before, many avirulent isolates of phytopathogenic species have proven their potential as biocontrol agents. For example, Aimé et al. [11] observed a downregulation of many defense-related genes in tomato after the inoculation of a non-pathogenic *F. oxysporum* isolate, while they reported an upregulation when a pathogenic isolate was subsequently inoculated. This phenomenon, called priming, provides the plant with an enhanced capacity to overcome more efficiently a pathogen attack [14]. The use of beneficial microorganisms in crops is becoming more appealing as they not only limit the development of pathogenic microorganisms by antibiosis, direct confrontation, rhizosphere colonization, and nutrient competition [11], but they can also trigger a defensive response in the plant or act as plant growth promoters [25]. Regarding the biological control of *Fusarium* wilts, promising results have been obtained using non-pathogenic isolates of *Fusarium* spp. [44]. The use of non-pathogenic isolates of *F. oxysporum* has been proposed as a viable strategy to prevent diseases in different crops like asparagus [45], bananas [20], and tomato plants [11] among others.

To summarize, the here presented results demonstrate that most defense-related genes in bean plants responded differently to pathogenic and low-pathogenic *Fusarium* isolates. On one hand, *F. oxysporum* F3 inoculation triggered a defense response that matched with other reports analyzing pathogen–plant interaction. On the other hand, plants inoculated with *F. oxysporum* F7 and *F. solani* F13 responded to this fungal presence in similar ways than those reported previously for plant–microbe interactions involving non-pathogenic isolates or biocontrol agents, indicating the putative hypovirulence of these isolates. The referred finding points out to the potential of non-pathogenic or low-pathogenic *Fusarium* isolates as biocontrol agents in this important crop. Nevertheless, while these results open up a new path towards the successful biological control of Fusarium wilts in common bean, further research using more fungal isolates and bean varieties, as well as in vivo and field trials, is needed to unravel the whole potential of this approach.

## 5. Conclusions

Out of the twelve *Fusarium* isolates evaluated, the presence of *F. oxysporum* F3 on the substrate showed reduced development of the bean plants, while the presence of *F. oxysporum* F7 and *F. solani* F13 on the substrate showed a lesser impact on bean plant development. Differences between these treatments were also observed in gene expression, correlating virulence and induction of defense-related gene expression. In this regard, *F. oxysporum* F3 triggered an upregulation of several defense-related genes in the plants, in line with previous studies with other phytopathogenic fungal isolates. In contrast, *F. oxysporum* F7 and *F. solani* F13 produced a general downregulation in the expression of defense-related genes compared to control plants. This behavior was similar to previous works that used both biocontrol agents and non-pathogenic fungal isolates to control fungal diseases. Therefore, the evidence from this study demonstrates that *P. vulgaris* interactions with low-pathogenic *Fusarium* spp. are comparable to those previously observed in other plant species, as well as to the interaction between bean plants and biocontrol agents or low-virulent isolates of other phytopathogenic fungal species. Hence, a door is open for further in vitro and in vivo research where low-pathogenic *Fusarium* isolates could be used to challenge pathogenic *Fusarium* isolates, finding new sustainable solutions to control fusariosis on bean crops.

## Figures and Tables

**Table 1:** *Fusarium* isolates used in the present study. All isolates are in the “Pathogens and Antagonists of the Laboratory Diagnosis of Pests and Diseases” (PALDPD) Collection, University of León, León, Spain.

Code	Identified As ^1^	% Identity	Bean Landrace
F1	*F. oxysporum*	>99%	Canela
F2	*F. oxysporum*	>99%	Canela
F3	*F. oxysporum*	>99%	Canela
F4	*F. oxysporum*	>99%	Pinta
F5	*F. oxysporum*	>99%	Canela
F6	*F. oxysporum*	>99%	Canela
F7	*F. oxysporum*	>99%	Pinta
F9	*F. oxysporum*	>99%	Riñón menudo
F10	*F. oxysporum*	>99%	Riñón menudo
F11	*F. oxysporum*	>99%	Riñón
F12	*F. oxysporum*	>99%	Pinta
F13	*F. solani*	>99%	Pinta

^1^*Fusarium* isolates were identified by TEF1 and ITS regions.

**Table 2:** Primer sequences used for RT-PCR analysis in common bean.

Gene	Functional Annotation	JGI Phytozome	Forward/Reverse
*Act11* ^2^	Actin-11	Phvul.008G011000	TGCATACGTTGGTGATGAGG AGCCTTGGGGTTAAGAGGAG
*Amintransf2* ^2^	Aminotransferase 2	Phvul.006G029100	TTCTTCCTTTTCTGCTCTTTCAA AGATGACAAGATGCAATGATTTTT
*Ukn1* ^2^	Unknown	Phvul.011G023200	ATTCCCATCATGCAGCAAAG AGATCCCTCCAGGTCAATCC
*CH5b* ^1^	Endochitinase precursor	Phvul.009G116500	CAGCCAAAGGCTTCTACACC TTGTTTCGTGAGACGTTTGC
*CNGC2* ^2^	Cyclic nucleotide-gated ion channel 2	Phvul.008G036200	ATTCAATTTGCTTGGAGACGTT ACAGTTTTATTGAAGGCCAGGA
*ERF1* ^2^	Ethylene-Responsive Transcription factor 1	Phvul.007G127800	CGCTCTCAAGAGGAAACACTCC TGAATCAGAAGGAGGAGGGAAT
*ERF5* ^2^	Ethylene-Responsive Transcription factor 5	Phvul.002G055700	GGCTCCAAGTGGATTGAGAAC TCAGAATCAGATAACTACAAAGCACAA
*GSTa* ^2^	2,4-D inducible glutathione S-transferase	Phvul.002G241400	AGGGAGTCACACTGGCTATGTT ATGTGCCATTTGCATTTTAGTG
*hGS* ^2^	Homoglutathione synthetase	Phvul.006G094500	GTGGCTATATGGTGCGTACAAA GAAACAAGAATGCATCTCCTCA
*HPL* ^2^	Hydroperoxide lyase	Phvul.005G116800	TCAAGGCTACATTTGTATTTCCA TGGTGCACATTTCTTAGTAGCAA
*IPER* ^2^	Peroxidase precursors	Phvul.009G215000	GGCAAGCATTATATGGTTGAAA GATGGCAACATCCATCACTTTA
*Lox2* ^2^	Lipoxygenase 2	Phvul.005G156700	ATGCAAGGCTAAAGAGATCCAA ATGGTGACAGGAGCTAAACACA
*Lox7* ^2^	Lipoxygenase 7	Phvul.005G156900	GAAGGCTTGACTTTCAGAGGAA AACACACGAGAAGATTCAACCA
*OSM34* ^2^	Osmotin-like protein	Phvul.002G155500	GAACGGAGGGTGTCACAAAATC CGTAGTGGGTCCACAAGTTCCT
*PAL1* ^2^	Phenylalanine ammonia-lyase	Phvul.001G177800	TGAGAGAGGAGTTGGGCACT TTCCACTCTCCAAGGCATTC
*PPO* ^2^	Polyphenol oxidase	Phvul.008G073200	GAAGACGATGATTTGCTGGTTA AAGAAACATTTTCCTTTGTGAAA
*PR1* ^1^	Pathogenesis-related 1	Phvul.003G109100	TGGTCCTAACGGAGGATCAC TGGCTTTTCCAGCTTTGAGT
*PR2* ^1^	Β-1,3 endoglucanase	Phvul.003G109200	GTGAAGGACGCCGATAACAT ACTGAGTTTGGGGTCGATTG
*PR3* ^2^	Basic Endochitinase B	Phvul.009G116600	TGGAGTTGGTTATGGCAACAA ATTCTGATGGGATGGCAGTGT
*PR4* ^1^	Pathogenesis-related 4	Phvul.006G102300	CGCAGTGAGTGCATATTGCT TGTTTGTCACCCTCAAGCAC
*PR16a* ^2^	Germin-like protein 8	Phvul.010G129900	GGCAGTCTCATGGTTATGGTTT GCATGCTCAAGTCTCAACACAT
*WRKY33* ^2^	WRKY transcription factors	Phvul.008G090300	TTTCACAGGACAGGTTCCAGC CCTTTGACAGAAATGACTGAAGGA

^1^ [25], ^2^ [24].
